# QTL Analysis for Bread Wheat Seed Size, Shape and Color Characteristics Estimated by Digital Image Processing

**DOI:** 10.3390/plants11162105

**Published:** 2022-08-12

**Authors:** Mian Abdur Rehman Arif, Evgenii G. Komyshev, Mikhail A. Genaev, Vasily S. Koval, Nikolay A. Shmakov, Andreas Börner, Dmitry A. Afonnikov

**Affiliations:** 1 Nuclear Institute for Agriculture and Biology, Faisalabad 38000, Pakistan; 2 Institute of Cytology and Genetics, Siberian Branch of the Russian Academy of Sciences, 630090 Novosibirsk, Russia; 3 Faculty of Natural Sciences, Novosibirsk State University, 630090 Novosibirsk, Russia; 4 Kurchatov Genomics Center, Institute of Cytology and Genetics, Siberian Branch of the Russian Academy of Sciences, 630090 Novosibirsk, Russia; 5 Leibniz Institute of Plant Genetics and Crop Plant Research, 06466 Seeland, Germany

**Keywords:** wheat, seed size, seed shape, seed coat color, phenotyping, candidate genes, QTLs

## Abstract

The size, shape, and color of wheat seeds are important traits that are associated with yield and flour quality (size, shape), nutritional value, and pre-harvest sprouting (coat color). These traits are under multigenic control, and to dissect their molecular and genetic basis, quantitative trait loci (QTL) analysis is used. We evaluated 114 recombinant inbred lines (RILs) in a bi-parental RIL mapping population (the International Triticeae Mapping Initiative, ITMI/MP) grown in 2014 season. We used digital image analysis for seed phenotyping and obtained data for seven traits describing seed size and shape and 48 traits of seed coat color. We identified 212 additive and 34 pairs of epistatic QTLs on all the chromosomes of wheat genome except chromosomes 1A and 5D. Many QTLs were overlapping. We demonstrated that the overlap between QTL regions was low for seed size/shape traits and high for coat color traits. Using the literature and KEGG data, we identified sets of genes in *Arabidopsis* and rice from the networks controlling seed size and color. Further, we identified 29 and 14 candidate genes for seed size-related loci and for loci associated with seed coat color, respectively.

## 1. Introduction

Bread wheat (*Triticum aestivum* L.) is a major staple crop. Millions of people depend on its production (https://www.fao.org/faostat/en/#data, accessed on 20 January 2022). This has led to an ongoing search for and study of genes that control wheat yield traits. Some of them are the characteristics of wheat seeds (size and shape) which have been shown to be related to seed weight [1,2,3,4]. Seed size and shape have also been shown to be related to flour quality and composition: small kernels can contribute to enhancing the bread-making quality of flour while having a detrimental effect on the milling yield [5]. To find genes that control these traits in wheat, QTL analysis is used. This analysis makes it possible to identify sets of markers that are associated with seed size or shape traits. Studies have shown that seed size and shape in wheat are controlled by a large number of loci located on almost all chromosomes [6,7,8,9,10,11,12,13]. Identification of these loci combined with molecular analysis can identify genes that are involved in controlling seed weight or size [14,15,16,17,18,19]. Based on genetic and molecular studies in both the model organism *Arabidopsis thaliana* and cereals, it is now established that seed weight is affected by multiple molecular and genetic aspects that lead to dynamic changes in cell division, expansion, and differentiation during seed development. Several important biological pathways contribute to seed weight, such as ubiquitination, phytohormones, G-proteins, photosynthesis, epigenetic modifications, and microRNAs [20,21]. Knowledge of the pathways controlling seed development in well-studied organisms allows the prioritization of candidate genes controlling these traits in wheat as well for their further study by molecular methods [15].

Another important characteristic of wheat seeds is the color of the shell. It characterizes the pigments and metabolites it contains. Purple and blue coloring of seeds is determined by the presence of anthocyanins. A yellowish color may be due to the presence of carotenoids. A reddish brown or dark brown coloration of the seeds is due to the presence of flavonoids such as proanthocyanidins and phlobaphenes [22]. Genetic control of color formation in both seeds and other plant organs is carried out by genes encoding the enzymes involved in pigment biosynthesis as well as regulatory genes [23]. For a number of pigments, these genes have been well studied; however, for some pigments, the molecular mechanisms of biosynthesis are still poorly understood [24].

The presence of pigments in the seed coat affects various technological properties of the seed [25] and is associated with antioxidant properties [26]. Therefore, varieties and lines with diverse seed coloration are of active interest in the food industry [27,28]. Seed shell color in wheat is also associated with important characteristics such as germination ability and pre-harvest sprouting (PHS). Red seeds are less susceptible to PHS [29]. QTL searches for seed color and PHS resistance are often simultaneously performed [30,31].

Recently, genotyping technologies have made great progress and include diversity array technology, genotyping-by-sequencing [32], and SNPs [33,34]. High-throughput genotyping can achieve high-density marker mapping [35]. This allows more QTLs to be obtained and, as a result, more accurately establish the molecular mechanisms controlling important plant phenotypic traits [8,32,33,36,37].

In the present work, we performed a SNP-based QTL search for seven traits of seed size/shape and 48 traits of shell color evaluated on the basis of digital image analysis on a set of 114 recombinant inbred lines (RILs) of the “International Triticeae Mapping Initiative” mapping population (ITMI/MP) and their parental plants.

## 2. Results

### 2.1. Analysis of the Seed Traits in ITMI Population

**Figure 1:**
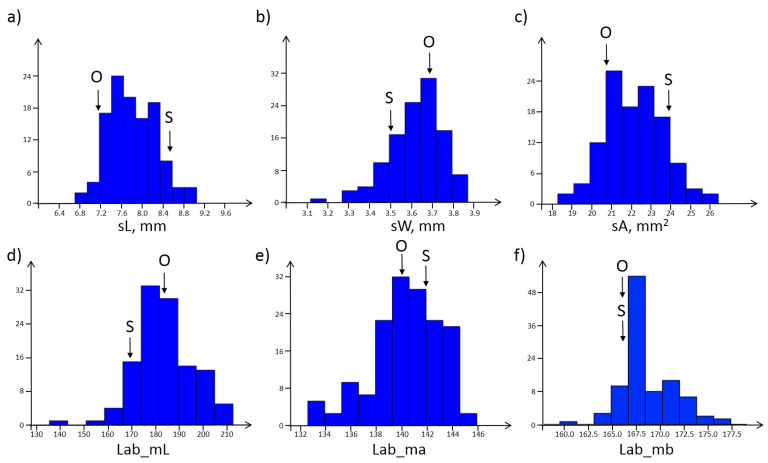
Distribution of six characteristics of seed size and color. The *X*-axis is the value of the characteristic, and the *Y*-axis is the frequency in the sample. (**a**) seed length, sL; (**b**) seed width, sW; (**c**) area of seed projection in the image, sA; (**d**) intensity of component L of Lab color space (lightness); (**e**) intensity of component a of Lab color space (redness); (**f**) intensity of component b of Lab color space (yellowness). The arrows show the characteristic values for the parental genotypes Opata (O) and Synthetic (S).

Figure 1 shows the distribution of six of the fifty-five seed characteristics in the ITMI population. Three of them characterize size (sL, sW, sA), and three characterize color (Lab_mL, Lab_ma, Lab_mb). The distributions were bell-shaped, and the hypothesis of normality was not rejected for the characteristics of seed length and area (Shapiro–Wilks test, *p* < 0.05), but for the width and color characteristics (Figure 1). Overall, the hypothesis of normality was not rejected based on this test for 22 of the 55 traits.

**Figure 2:**
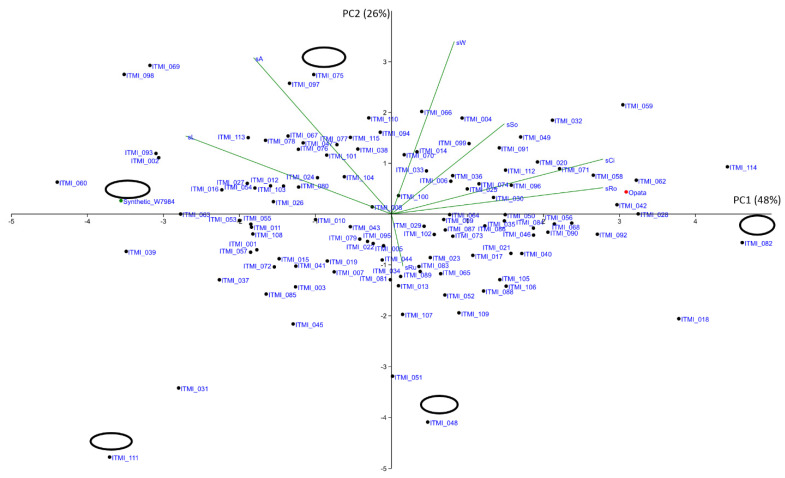
PCA biplot of seed size and shape of ITMI/MP performed using seed 7 coat traits. Ellipses represent seed size and shape for some contrast genotypes in the same scale. Parent genotypes are shown by green (Synthetic_W7984) and red (Opata) dots. PC1, PC2 axes denote principal components 1 and 2, percentage of variance explained shown in parentheses.

**Figure 3:**
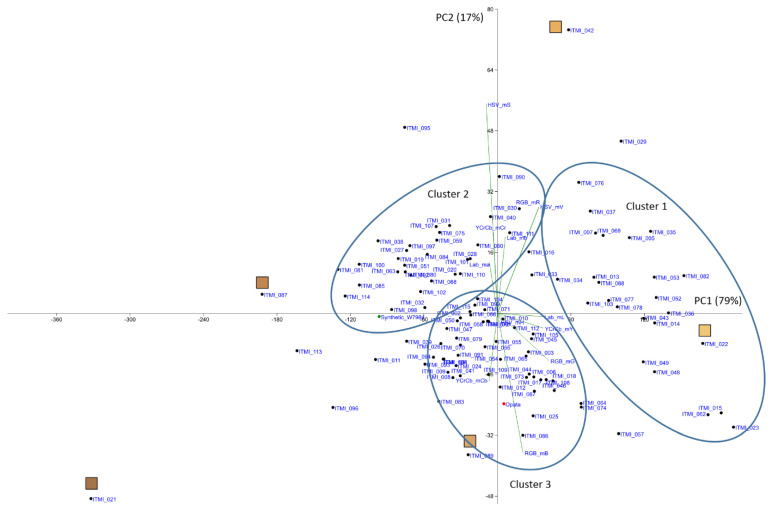
PCA biplot of seed coat color of ITMI/MP performed using seed coat traits (mean values for 12 color components of four-color spaces). Color bars represent coat color for some contrast genotypes. Parent genotypes shown by green (Synthetic_W7984) and red (Opata) dots. PC1 and PC2 axes denote principal components 1 and 2. Explained percentage of variance shown in parentheses. Three clusters of genotypes shown by ellipses.

In order to visualize the distribution of genotypes in the space of the considered traits, we performed principal component analysis for the traits of shape/size (all seven traits) (Figure 2), color (12 traits of average values of color components in four-color spaces) of seeds independently (Figure 3) and all these 19 traits simultaneously (Figure S1). From the diagram of the principal components in the size/shape feature space, we see that the first component characterizes the roundness of the seeds and is correlated with circularity. The second component characterizes seed size (most related to width and area). The most characteristic genotypes are ITMI_082 (the most rounded seeds), ITMI_075 (large area), ITMI_048 (small area), and ITMI_111 and Synthetic_W7984 (most elongated). The second parental genotype is located in this diagram on the far-right side of the diagram, close to the *X*-axis, i.e., it has a rounded seed shape. It is difficult to distinguish any noticeable clusters in this diagram: it is a cloud with some distant genotypes. Notably, this cloud is more sparce in the upper half-plane (PC2 > 0) and compact in the lower half-plane (PC2 < 0). 

The diagram of the principal components in the color feature space shows that the first component primarily characterizes the lightness of the shell (correlates with Lab_mL and YCrCb_mY). The second component characterizes seed color saturation and reddish shade (positively correlates with HSV_mS, RGB_mR, and Lab_ma characteristics). The most characteristic genotypes are ITMI_2 (the lightest shell), ITMI_042 (the most saturated color), ITMI_088 (the palest shell hue), and ITMI_021 and ITMI_087 (the darkest shell hue). Three clusters can be distinguished on the plot (Figure 3). Seeds from plants of the first cluster have a lighter color (large values of PC1), with a large part of them having a more reddish color (positive values of PC2). Seeds from plants of the second cluster have darker (negative values of PC1) and more reddish color (positive values of PC2). Seeds from plants of the second cluster have less reddish color (positive values of PC2 and PC1 values dispersed about 0 value). Interestingly, parent genotypes Synthetic_W7984 and Opata fall into distinct clusters on the plot (Cluster 2 and 3, respectively).

The diagram of the principal components in the seven size/shape and 12 color feature space (Figure S1) shows that the first component characterizes the color of the seed shell: the negative values are characteristics of reddish color (PC1 positively correlates with Lab_mb and negatively correlates with Lab_ma). The second component characterizes seed size/shape (positively correlated with roundness, sRo, and circularity, sCi and negatively correlates with area, sA, and length, sL). No clear clusters were detected in this plot for genotypes.

### 2.2. QTL Analysis

**Figure 4:**
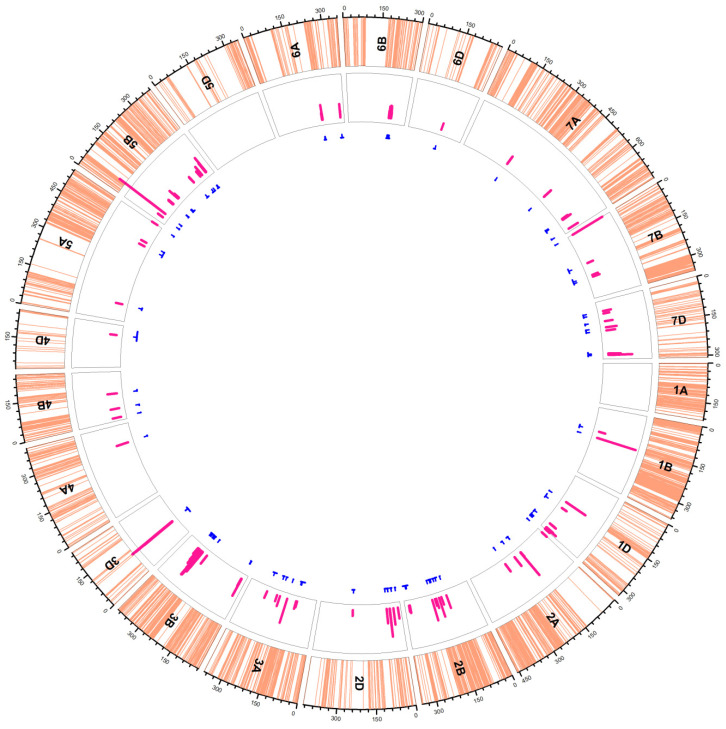
Distribution of additive QTLs (blue lines in the inner circle). Light orange lines in the outer track indicate the SNP positions on each chromosome. Pink bars in the second circle indicate the LOD values of QTLs. The blue lines under the track circle indicate the confidence interval of QTLs with small vertical lines pointing to the peak position of QTL. For details, see Table S1.

Genetic analysis of the three characteristics of seed size (sL, sW, and sA), four characteristics of shape (sCi, sRo, sRu, and sSo), and 48 characteristics of color (12 characteristics each of RGB, HSV, L *a *b, and YCrCb) revealed a total of 20, 22 and 170 QTLs (212 in total) (Figure 4, Table S1), correspondingly, on all the chromosomes of wheat genome except chromosomes 1A and 5D. The number of QTLs varied from one (characteristics: HSV_mS, HSV_dCH_2, HSV_dCS_2, and HSV_dCV_2) to ten (characteristic: sA) for one single trait. The majority of the traits yielded three (13 characters) to four QTLs (19 characters). 

Among chromosomes, the highest number of QTLs was observed on chromosome 3B (46 QTLs), followed by chromosomes 3D and 6B with 34 and 27 QTLs, correspondingly. Chromosome 5B carried 15 QTLs, and the chromosome 2B carried 14 QTLs, whereas chromosome 7A carried 12 QTLs. This was followed by chromosome 1D with 11 QTLs. Chromosomes 3A and 7D carried nine QTLs each, and chromosome 6A carried seven QTLs. Chromosomes 6A and 2D carried seven and six QTLs, respectively. Five QTLs resided on each of chromosomes 2A and 4B, whereas four QTLs resided on each of chromosomes 5A and 7B. On the other hand, two QTLs were detected on each of chromosomes 1B and 6D. Finally, chromosomes 4A and 4D carried one QTL each. In terms of groups, group 3 chromosomes carried the highest number of QTLs (89), whereas group 4 chromosomes carried the least number of QTLs (five). Group 6 chromosomes carried 36 QTLs, whereas each of group 2 and 7 chromosomes carried 25 QTLs. On the other hand, group 5 chromosomes carried 19 QTLs, and group 1 chromosomes carried 13 QTLs. 

**Figure 5:**
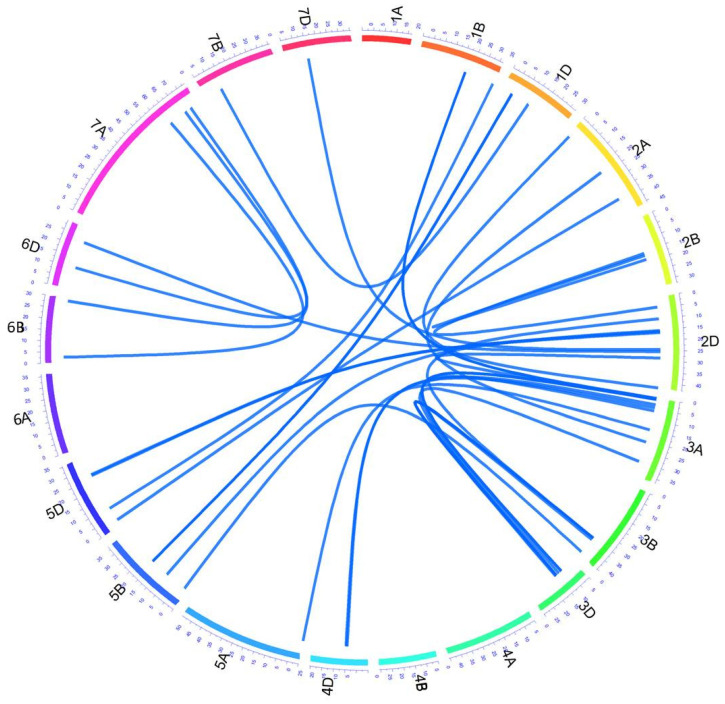
Epistasis QTL network in the ITMI/MP. Outer circular plot represents the hexaploid genome arranged in chromosomes (chrs) 1–21 (1A–7D) in clockwise direction. Numbers on colored outer circle represents cM on respective chrs. Blue-colored connections represent epistasis QTLs controlling different traits. For details, see Table S2.

Additionally, we were able to detect 34 pairs of epistatic QTLs controlling at least 22 characters in our RILs, with five characters under the influences of more than one pair of epistatic QTLs (Figure 5, Table S2). These QTLs involved all the wheat chromosomes except chromosomes 1A, 4A, 4B, and 6A. The most frequently involved chromosome was 3D (12 times), followed by chromosomes 3A (11 times), and 3B (nine times). Chromosome 2D was involved six times, whereas chromosomes 5B and 5D were involved four times each. Chromosomes 1D, 2B, and 7A were involved three times each. Two times involvement was observed for chromosomes 1B, 2A, 4D, 6B, and 6D, whereas the chromosomes 5A, 7B, and 7D were only involved one time. 

### 2.3. Analysis of the Similarity of Traits by QTL Location

We observed remarkable overlap between QTL locations for different traits. For example, two QTLs related to seed shape, *Q.sCi-2B ^c^* (circularity) and *Q.sSo-2B ^c^* (solidity), were located in the same position 129 of chromosome 2B. The 3B chromosome has loci with multiple traits associations: position 39.179 (two traits of size), position 298.179 (seven color traits), position 299.179 (twelve color traits), position 300.179 (two color traits), position 306.179 (ten color traits), position 308.179 (two color traits), position 311.179 (two color traits and one shape trait, rugosity), and position 324.179 (three color traits). This is not surprising because our parameters estimated from images represent various quantifications of the same biological seed property (i.e., seed weight, pigment concentrations in the coat, etc.). This suggests that the set of our characteristics is degenerating and that many of them, in fact, are controlled by the same genes. 

**Figure 6:**
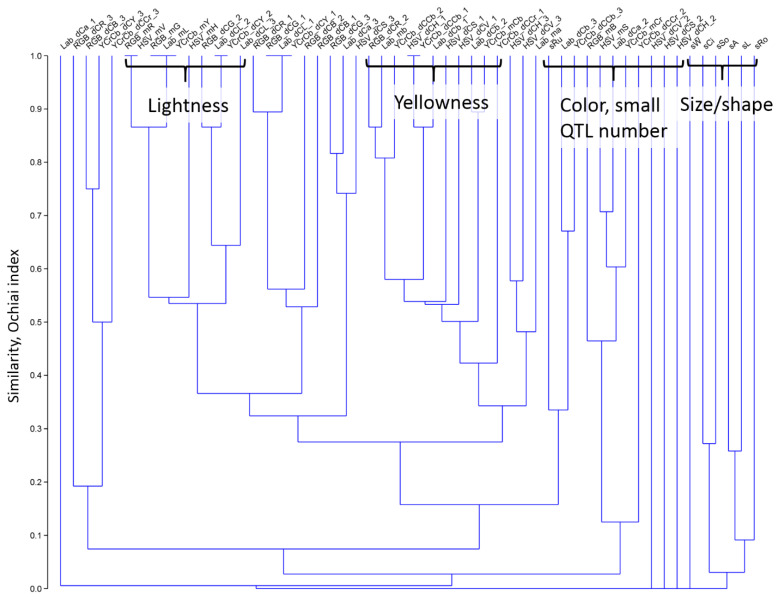
The similarity tree for seed traits obtained by the degree of the overlap between their QTL locations. The vertical axis represents the similarity measure based on the Ochiai index (Y axis). Leaves correspond to seed traits described in [1] ( for trait abbreviation, see Section 4.2*. Quantitative Characteristics of Seed Shape, Size and Color*). Groups of traits with strong overlapping of the QTL locations are shown by curly brackets.

To evaluate the similarity of various traits under analysis, we hierarchically clustered them by the degree of the overlap of QTL locations (Figure 6). The tree diagram demonstrates several interesting features. Firstly, size/shape characteristics (right part of the tree) are clearly separated from the color traits (with the exception of rugosity, sRu). Secondly, some traits with a small QTL number (one to two) are also separated from other traits. Thirdly, a remarkable number of traits related to yellowness form a large cluster. Finally, traits related to the seed lightness (Lab_mL, HSV_mV, and YCrCb_mY) fall in the same cluster, and their QTLs are highly overlapped. Other color traits are irregularly dispersed on the tree within the large cluster of color traits. 

### 2.4. Gene Prioritization

A search for orthologous groups for the eight pathways of pigment biosynthesis and their precursors identified 307 KEGG orthologs involved in these processes (Table S3). A review of the literature [18,19,38] identified 155 *Arabidopsis* and 42 rice genes involved in the molecular processes of seed development (Table S3). Of these genes, 193 were found to have sequence identifiers in the KEGG database, and 109 of them were associated with KEGG orthologous groups (Table S4).

For prioritization of genes, we used 48 highly significant QTLs with LOD > 3 for which marker positions were identified in the wheat genomic sequence (Table S5). On this basis, we identified 2787 unique genes localized to marker-limited sites. Of these, 1422 genes associated with seed size/shape, and 1365 genes associated with seed color. After filtering by expression level, 823 genes associated with seed color remained (Table S6). For these sets of genes, we performed KEGG orthogroup assignment using BlastKOALA and KofamKOALA services. For 464 genes associated with size trait loci and 321 genes associated with color traits, such orthogroups were found. 

For 29 genes from the seed size-related loci, we found a match within the orthogroup list obtained based on the analysis of the literature data (Table 1). Eleven genes identified in this way belong to regulatory proteins (transcription factors EREBP, HD-ZIP, and MYBP; loci on chromosomes 3A, 2B, 2D, and 7D). Six genes belong to translation initiation factors (ELF2C; loci on chromosomes 2B and 7D). Five genes relate to enzymes associated with ubiquitination processes (loci on chromosomes 2D). Four genes have chitinase activity (locus on chromosome 7D), two genes with cytokin dehydrogenase activity (chromosomes 3A and 7D), and one aarF domain-containing kinase gene (chromosome 7D). 

For genes from the loci associated with seed coat color, 14 found a match with the orthogroups of the metabolic pathways of the KEGG database related to pigment biosynthesis (Table 2). Eight genes were involved in the phenylpropanoid biosynthesis (loci on chromosomes 3A, 3B, 6A, and 6B). Two genes were involved in the carotenoid biosynthesis pathway (loci on chromosomes 2A and 6A), and one gene each was involved in flavone and flavonol biosynthesis, flavonoid biosynthesis, tryptophan metabolism, and terpenoid backbone biosynthesis.

## 3. Discussion

### 3.1. Using Digital Image Analysis for QTL Identification

Based on the analysis of digital images, we identified QTLs associated with quantitative seed characteristics in wheat. With the development of modern phenotyping technologies [39,40], such approaches are increasingly being used [7,8,9,10,11,41]. Modern digital cameras and image processing algorithms have made great progress; they allow us to estimate even small differences in the color characteristics of seeds, their shape and size with high accuracy. In addition, these approaches have one interesting feature: the use of a large number of quantitative characteristics that are essentially derived from the same biological trait of the plant. For example, seed shape and size could be described as the sets of elliptic Fourier components [41] or virtual curves [42,43]. Components of various digital spaces [44,45] represent seed coat color. In the case when quantitative traits are derived from the same biological trait, we can assume that they will be associated with the same loci. Williams and Sorrels [11] used QTL for a set of seed size and shape characteristics derived from the developed seed image in two projections (elliptic Fourier components) as well as length, width, and thousand-kernel weight (TKW). They studied two populations, one of which, SynOpDH, was derived from crosses of the same parents used to obtain the ITMI population, and the other Cayuga × Caledonia was a doubled-haploid mapping population (C × C). Thirty-one loci were identified for the SynOpDH population which controlled from one to four traits per locus. Thirty loci were identified for the C × C population which also controlled from one to four traits per locus.

From our results (Figure 6), it is apparent that many color trait loci overlap between each other but not with the QTLs of size/shape (Table S1; chromosomes 3B, 3D, and 6B). The exception is rugosity which reflects the roughness of the shell and is probably associated with color distortions at the seed boundary on the image background. On the one hand, these features reflect the degeneracy of the evaluated traits which indicates their redundancy. On the other hand, the location of several loci related to color traits in the same region may indicate a more reliable identification of the association of the locus with a particular trait. Using many digital representations of the same trait looks redundant and confusing. The reasonable step would be to select a single or a few numerical characteristics that are most efficient in the identification of QTLs. We believe that various numerical representations of the same biological trait are useful and allow the evaluation of its subtle details. Many traits with QTL in the same locus can support its significance.

### 3.2. Identification of QTLs Associated with Seed Features

Our analysis allowed us to identify a number of QTLs associated with wheat seed characteristics, shape/size, and shell color. Such analyses have long been intensively conducted based on both QTL and GWAS investigations [8,11,41,46,47]. Reference [11] investigated three-dimensional characteristics of seed size and shape based on the analysis of images of seed obtained in two projections and the use of Fourier analysis-based descriptors using two populations, one of which was SynOpDH. They found a QTL that affects a number of shape characteristics. For seed length, eight QTLs were found for chromosomes 2A, 2D, 4B, 5A, 5B, 6A, 7A, and 7D. In our work, we detected a smaller number of QTLs for this trait located on the chromosomes 2A, 2D, 3B, 5B (2 QTLS) and 7D. For seed width, three QTLs on chromosomes 2A, 5A and 6A were detected by [11], whereas we detected four QTLs for this trait located on the chromosomes 1D, 2D, 3B, and 4D. This anomaly could be due to the use of data from several different environments in many years by reference [11]. 

Reference [47] previously analyzed 92 accessions from the ITMI population for a large number of traits, including such traits as TKW and kernel color (KC), in different locations and years. We did not find any overlap of QTL for TKW with the traits characterizing seed size and shape in our work. For seed color, 15 QTLs were reported by [47]. A comparison of our results with those from this work showed that of the 15 QTLs, one exactly matched the one found in our work. This is Q.KC_Pu07-3B [47] bounded by markers AX-94979462 and IAAV6088 and located in our work on chromosome 3B at position 306.179 (Table S1). In our work, several QTLs associated with seed shell color characteristics correspond to this locus (see marker HSV_dCH_1-3Bm (Table S1) and also listed in Table 2.) It is also interesting to note the QTLs Q.KC_Mo07-3D and Q. KC_Mo08-3D [47] bounded by markers D_GDS7LZN02IJRXZ_309 on the left and CAP12_c2615_128 on the right located on chromosome 3D at 76 cM. In our work, we found a series of color-related QTL localized on chromosome 3D at position 100–102, bounded by markers CAP12_c2615_128 on the left and BS00067163_51 on the right. Thus, the QTLs from our work and that of reference [47] are in the vicinity on the chromosome. For the other QTLs associated with color, we found no coincidence. For example, reference [47] identified five QTLs associated with shell color on chromosome 5A. However, in our work, only two loci at other positions on this chromosome were associated with color. 

Among the loci associated with color, the site on chromosome 3D (ar 100 cM) bounded by the markers *CAP12_c2615_128* and *BS00067163_51* is perhaps the most interesting. As indicated above, it is located next to the color QTL identified in [47]. In our work, 34 different traits characterizing seed color are associated with it. All of them are color parameters in various color spaces. Our analysis allowed us to localize the physical coordinates of this site on chromosome 3D: 573.6–580.8 Mbp according to IWGS v2.0 genome annotation (Table S5). Interestingly, reference [48] recently performed the analysis of the *PHS-3D* QTL associated with seed resistance to pre-harvest sprouting for a population of synthetic hexaploid wheat [49]. It turned out that on the physical map this region is located on chromosome 3D at positions 571.9–574.3 Mbp which overlaps with the physical localization of the QTL we identified. Reference [48] also showed that plant genotypes susceptible to pre-harvest sprouting are characterized by a ~2.4 Mbp deletion involving 20 genes in this region of the genome. It turned out that the gene encoding the transcription factor Myb10-D, which confers resistance to pre-harvest sprouting by activation of flavonoid and abscisic acid biosynthesis pathways, was located in this region. Note that plants that do not contain deletions in this region and are resistant to pre-harvest sprouting have reddish/brown coloring of the seed shell.

### 3.3. Epistatic QTLs

In our work, we identified several QTLs whose contribution to the trait are non-additive. Currently, there are only a few examples of epistatic QTLs analysis in wheat [46,50,51,52]. We found 34 QTL pairs exhibiting epistatic interactions. Our results show a predominance of epistatic QTLs for color features (30 pairs of QTLs). One pair of QTL each was identified for the area, width, rugosity, and solidity of seed. These results demonstrate a possible interaction of genes located at different loci in the formation of color traits.

Epistatic QTLs for yield, flour color, and seed weight traits were investigated for the RIL population of durum wheat [51]. QTL epistatic interactions on chromosomes 1A and 1B and chromosomes 5B and 7B were determined for thousand-seed weight. Reference [46] analyzed yield traits including 1000-seed weight, seed length, and seed width of bread wheat in the RIL Chuannong18 × T1208 population. Epistatic QTLs were found for 114 QTL pairs, including 10 for seed length, 17 for seed width, and seven for 1000-seed weight. The authors noted the complex nature of the effect of epistatic interactions on the seed properties. Thus, more epistatic pairs for geometric seed traits were identified in these works compared to ours. However, in our work, the most intense epistatic interactions were shown for seed shell color, a trait not reported in [46]. Interestingly, QTL pairs that are localized on chromosomes 3B (position 306) and 3D (position 100), which are also characterized by a large number of additive QTLs, are often represented. This again indicates the importance of these regions for the formation of seed shell color in wheat.

### 3.4. Gene Prioritization

Our analysis allowed us to identify a number of candidate genes associated with seed size/shape and their color, based on bioinformatics analysis and annotation of genes according to data in the literature and the KEGG database. We identified eight loci associated in the genome with seed size/shape traits for which we found 28 orthologous genes involved in gene networks controlling these traits. Some of genes are transcription factors (EREBP, HD-ZIP, and MYB) that may be involved in the regulation of seed growth and development. In particular, transcription factors associated with the response to ethylene (EREBP) are known to be involved in the determination of seed size, seed weight, and accumulation of seed oil and protein in *A. thaliana* [53]. Reference [54] identified two transcription factors from the AP2/EREBP family, TaPARG, located on 2A and 2D chromosomes of wheat which regulate several yield-related traits, including seed weight. 

Several genes represent families of enzymes related to ubiquitin modification (E3 ubiquitin–protein ligase, ubiquitin thioesterase protein OTUB1). Ubiquitins and related enzymes are known to play an important role in seed development by controlling cell proliferation [55]. For example, genes of the E3 ligase family are involved in amylose biosynthesis in wheat [56]. The *TaGW2-6A* gene from this family is shown to control seed size [57]. 

Another type of enzyme that was frequently found among the candidates we identified are endochitinases. These are enzymes involved in defense against pathogens such as bacteria or fungi in seeds [58,59]. However, this is not their only role in seed formation and function. It has been shown from proteomic data in rice that *chitinase 14* interacts with the *GW2* (*RING-type E3 ubiquitin ligase*) gene. It was also shown that *GW2* controls seed size through the regulation of chitinase 14 and phosphoglycerate kinase levels or activities [60]. Other genes that we detected (cytokinin dehydrogenase, aarF domain-containing kinase, and eukaryotic translation initiation factor 2C) may also be associated with seed development in wheat [20].

For QTLs associated with seed color, we also found a number of possible candidates among genes encoding enzymes of plant pigment biosynthesis pathways. On chromosome 2A, we found several genes that are involved in plant pigment biosynthesis. Among them, one gene, which we annotated as NCED, is involved in the carotenoid biosynthesis pathway. In rice, mutants of this gene lead to changes in pericarp seed coloration [61]. The expression of this enzyme is controlled by abscisic acid [62], and *NCED* is also involved in ABA biosynthesis [48]. The functions of this gene in seed development are well known [63]: it is an important regulator in seed development, in the zygotic embryogenesis, and dormancy. Another gene related to carotenoid biosynthesis that we found among the primate genes is *CYP707A ((+)-abscisic acid 8’-hydroxylase*) (Table 2). Its functions are closely related to the *NCED* gene, and its participation in the same processes related to seed development has been shown [63]. Interestingly, two of these genes are located near loci associated with seed coat redness (*Q.YCrCb_dCCr_1-2A.3* and *HSV_dCH_3-6At/Lab_ma-6At*).

We found two genes that are involved in the flavonoid biosynthesis pathway (Table 2) that provide different coloration of seeds in cereals [64,65]. These include the homologue FG2 (*flavonol-3-O-glucoside L-rhamnosyltransferase*), for which mutations result in a phenotype with seed color change in soybean [66]. *Shikimate O-hydroxycinnamoyltransferase* has been shown to be elevated in expression in wheat plants with high seed antioxidant activity [67].

In the QTL region of *Q.Lab_dCb_3-3Ai*, we found two genes involved in phenylpropanoid biosynthesis. They both encode *4-coumarate--CoA ligase*. This enzyme catalyzes the conversion of p-coumaric acid to p-coumaroyl CoA, which further serves as a source of biosynthesis of both lignin (a structural component of the seed shell) and flavonoids. In transcriptome-wide association studies in *Brassica napus*, 4CL expression during seed development was shown to positively correlate with seed coat content, i.e., the fraction of seed mass attributable to the coat [68]. Interestingly, in gene expression analysis in *B. napus* plants with brown seed coloration, the expression level of genes encoding this enzyme was higher than in plants with yellow coloration [69].

Modern genomics advances in wheat genome sequencing and genetic marker technologies allow QTLs to be linked to the physical coordinates of the wheat genome. Such analysis is now an important complement to QTL identification [70,71,72]. The genes we have identified as possible candidates associated with seed size/shape and color formation in the ITMI/MP can be further investigated in more detail using genetic and molecular methods to establish the mechanisms controlling these important traits.

## 4. Materials and Methods

### 4.1. Materials

We studied seeds from 114 accessions of the well-known ITMI/MP of bread wheat (*T. aestivum* L.). The ITMI mapping population was obtained by pollination of the *T. aestivum* (var. Opata 85) flower with the pollen of the synthetic hexaploid spring wheat W7984 [47]. Plants of each genotype were grown in season in 2014 on the experimental fields of IPK in Gatersleben, Germany. 

### 4.2. Seed Imaging Protocol and Image Processing

Seeds were imaged in March 2020. We supposed that the storage time affected seed traits for different genotypes in the same manner. The imaging of seeds was performed according to the protocol described earlier [1]: seeds were scattered in an amount of up to 20 pieces on the table on a white sheet of A4 paper. A ColorChecker color calibration card (x-rite ColorChecker^®^ Classic Mini, X-Rite, Grand Rapids, MI, USA, https://xritephoto.com/camera; accessed on 20 January 2022) was placed in the image area and used for color correction and obtaining image scale. The lighting was adjusted to avoid shadows. Images were taken with a digital camera Canon EOS 600D equipped with a Canon EF 100 mm f/2.8 Macro USM lens and saved in files in JPG or PNG format. Examples of images are shown in Figure S2. Digital image processing was performed using the SeedCounter application for desktop PC [73] with color analysis capabilities [1]. We used two images per genotype for our analysis: 15 and 5 seeds. Splitting was initially used to check for reproducibility of the evaluated trait values. No significant differences between mean values of the seed traits were observed between these replicates according to an *F*-test (results not shown). Therefore, we used the average values of the images of 20 seeds from two replicates as input for QTL analysis.

### 4.3. Quantitative Characteristics of Seed Shape, Size, and Color

The analysis of digital images for each seed yielded a set of 55 quantitative characteristics described earlier [1]. Size was defined by seed length (sL), width (sW), and projected area (sA). Seed shape characteristics included circularity (sCi), roundness (sRo), rugosity (sRg), and solidity (sSo).

The circularity and roundness indices reflect how close the shape of a contour is to a circle but are calculated differently. Circularity is a measure of the similarity of a 2D figure to a circle [43]. For objects with rugged contours, the closeness of the shape to a circle is more correctly described by the roundness parameter since this value does not depend on the roughness of the contour line. This index is calculated as the ratio of the area of the shape (area) to the square of the length of the major axis [40]. For a shape other than a circle, the index takes values less than unity. The rugosity index (sRg) is defined as the ratio of the contour perimeter to the convex perimeter [43]. The index of solidity (sSo) is the ratio of the contour area to the area of its convex hull [74]. 

To describe the color characteristics of the seeds, we used a color representation in the form of four-color spaces: RGB, HSV, Lab, and YCrCb [44,75,76]. Each of them represents color as three components. The component values of one space can be obtained by transforming the component values of the other. The color features included two types of descriptors, which were independently calculated for each color space.

The first type of descriptors: mean values of component intensities for seed pixels. To calculate them, the mean and standard deviations of intensities for each of the color component channels were first estimated, then the pixels whose intensities differ from the mean by more than three standard deviations were excluded from the analysis. The mean value was calculated for the remaining pixels and used further. The descriptors of the average component values are indicated by a small letter m. For example, for the RGB color space, these are the three parameters: RGB_mR, RGB_mG, and RGB_mB. For other spaces, the indications are similar.

The second type of descriptors are dominant seed colors. These descriptors provide an illustration of representative colors in an image or its region [77]. To determine dominant colors, all seed pixels were grouped by color similarity into three clusters. The clusters were ranked by the number of pixels they contained. In each of the three clusters, the values of the three color components for the centroid were determined. This procedure was performed for each color space and resulted, respectively, in nine color descriptors. For example, for the RGB space, these are RGB_dC*j*_*i* parameters, where *j* = 1,2,3 is the color component designation, and *i* = 1,2,3 is the number of the dominant cluster. For example, the RGB_dCR_1 parameter is the R component for the first dominant color in the RGB space. The use of three dominant colors allows for a more accurate estimation of the shades of seed coloration.

As a result, three size characteristics, four shape characteristics, and 48 color characteristics were determined for each seed. Characteristics were calculated for each seed of the 114 wheat genotypes. The mean values for the genotype were estimated and used in the QTL analysis. 

### 4.4. Statistical Analysis 

To get an idea of the similarity of genotypes in the space of seed traits, we used the principal component method implemented in the PAST program [78].

### 4.5. Genotyping and QTL Analysis

Fresh flag leaves were used for the DNA extraction for the purpose of genotyping which was performed using Illumina (San Diego, CA, USA) Infinium technology. An optimized array (wheat 20K Infinium SNP array) was used. This array is the refined version of the 15K chip [79] of 90K iSELECT SNP-chip as previously reported [80]. To make it more informative, 5385 markers from the 35K Wheat Breeders Array [81] were also added. All sequences of the markers, a complete genetic map and the list of 92 RILs with genotypic data are available in reference [47].

To capture the variance explained by the molecular markers such as SNPs mapped to any genome, different methods were proposed (such as single marker analyses, interval mapping, and composite interval mapping) and implemented in different computer programs (*Qgene*, *QTL Cartographer* and *PLABQTL*) [82] which have successfully been used to detected QTLs for various traits in wheat including seed-related traits such as seed longevity [37,83,84]. In light of the limitations of the above-mentioned methods, a more refined method known as “inclusive composite interval mapping” was proposed and implemented in the *QTLIciMapping 4.2.53* (http://www.isbreeding.net/ (latest released in September 2019, accessed on 2 February 2022) which is considered as the most modern method of QTL detection [47]. We have recently detected several QTLs for *Fusarium* head blight [85] and seed longevity [50] in wheat and germination-related traits in tobacco [86] by applying the *QTLIciMapping* tool. Therefore, we convened the *IciMapping 4.2.53* to detect the putative additive QTLs of the traits under consideration by applying the inclusive composite interval mapping (ICIM) command where 1.0 cM was the walking speed. An LOD score of >2.0 ≤3 was applied to detect QTLs as significant and >3.0 as highly significant [87]. 

In order to discover digenic epistasis QTLs to find clues for latent variation, the ICIM-EPI command was used where LOD was kept at 5.0 cM. Here, the epistasis QTLs with LOD ≥5 and explaining ≥5% phenotypic variance were reported. All QTLs were assigned names according to the rules set out in the Catalog of Gene Symbols [88]. All additive and epistasis QTLs were visualized using the “circlize” package in R [89]. 

### 4.6. Seed Traits Similarity by QTL Location

Preliminary analysis of QTLs demonstrated that loci for several traits often overlap. In particular, the same locus may associate with several characteristics of the seed shell color. In this regard, we decided to analyze the similarity of traits by their localization in the genome. To do this, we compiled a list of loci with which they were associated for each of the 55 features. Based on the overlap of the list of these loci, we calculated the Ochiai index [90] for each pair of features. This index was suggested for ecological studies to estimate associations between species and groups of sites representing habitat types. In our work, the index reflects the degree of overlap of the lists of loci between two traits. The greater the similarity between sets of loci for two traits, the greater the index value. It equals 1 when loci are identical and 0 when there is no loci overlap. Based on this measure, we performed clustering of features in the PAST [78] package and built a tree of the trait similarity.

### 4.7. QTL Gene Prioritization

In order to identify possible candidate genes associated with seed traits, we prioritized them based on several conditions and using the gene annotation provided in the KEGG database [91]. The analysis included loci for which the LOD value exceeded three and consisted of several steps. 

In the first step of the analysis, we determined the physical localization of the markers by aligning their sequences to the IWGS 2.1 wheat genome assembly sequence [92]. Genome sequence and annotation data were obtained from URGI (https://urgi.versailles.inra.fr/download/iwgsc/IWGSC_RefSeq_Assemblies/v2.1; accessed on 10 January 2022). We considered “high confidence” gene annotations only. Marker sequences were obtained from reference [80] and the Gramene marker database (https://archive.gramene.org/markers/; accessed on 10 January 2022) [93]. Marker sequences were aligned using BLASTn of the BLAST+ package [94] using e-value = 1 × 10^−17^ (other parameters were set by default). This allowed us to search similar sequences with length above 50 nt and avoid noise. Thus, for each of our selected QTLs, we obtained a list of IWGS 2.1 wheat genome annotation genes. Note that it was not possible to determine the physical boundaries of the QTLs for several loci because, for one of the sequences, the alignment did not occur on the chromosome corresponding to the marker. 

Since plant pigments can be synthesized in various tissues and organs, when prioritizing genes for seed color QTL, we additionally filtered genes by expression level in the seed. For this purpose, we used the expression data presented for wheat in the expVIP database [95]. Data in text format were downloaded from URGI (https://urgi.versailles.inra.fr/download/iwgsc/IWGSC_RefSeq_Annotations/v1.1/iwgsc_refseqv1.1_rnaseq_mapping_2017July20.zip; accessed on 10 January 2022). We used data from RNA-seq experiments in which the column “High level tissue” contains “seed”. We selected genes as expressed if their TPM ≥ 1 in these experiments. To perform filtering, we developed scripts in Python, taking into consideration the Gene ID conversion between annotation ver. 2.1 (genome) and 1.2 (transcriptome).

In the second stage of analysis, we generated a list of orthologous protein groups from the KEGG database [91], which are associated with the formation of the traits of seed size and color. It is well known that the color of the seed shell is determined by the presence of specific plant pigments in it [22]. Therefore, we selected orthogroups involved in KEGG pathways of the biosynthesis of these pigments and a number of their precursors. The list of such pathways includes tryptophan metabolism (map00380), terpenoid backbone biosynthesis (map00900), carotenoid biosynthesis (map00906), phenylpropanoid biosynthesis (map00940), flavonoid biosynthesis (map00941), anthocyanin biosynthesis (map00942), isoflavonoid biosynthesis (map00943), and flavone and flavonol biosynthesis (map00944). We obtained 307 KEGG orthogroups for these pathways. 

Seed size depends on a multitude of biological processes occurring at the molecular level, including protein ubiquitination, response to hormonal signals, protein biosynthesis and transport, etc. Therefore, it was not possible to isolate the pathways corresponding to these processes based only on their description in the KEGG database. However, the genes involved in seed development have been fairly well experimentally studied in *A. thaliana* and rice (*Oryza sativa*). Therefore, we used three recent literature reviews describing the molecular processes of seed development in *Arabidopsis* and rice [20,21,38]. We combined set of genes from three papers and removed duplicated IDs. During compilation, we converted gene IDs from reference [20] from RAP to MSU format using the “ID converter” tool at the website of the OryzaExpress database ([96]; http://bioinf.mind.meiji.ac.jp/OryzaExpress/ID_converter.php; accessed on 20 January 2022). We identified KEGG orthogroups for the selected genes and used them for our analysis. 

The assignment of KEGG orthologous groups to wheat genes by their sequence was performed using BlastKOALA [97] and KofamKOALA [98] tools. The orthogroups were assigned to genes by at least one of the methods. We prioritized genes whose orthogroups were in the lists associated with traits of seed coat color and size. 

## 5. Conclusions

A QTL search for seven traits of seed size/shape and 48 traits of coat color evaluated on the basis of digital image analysis of the ITMI/MP identified 212 additive and 34 pairs of epistatic QTLs on all the chromosomes of wheat genome except chromosomes 1A and 5D. The number of QTLs varied from one to ten for one single trait. The majority of the traits yielded three to four QTLs. We demonstrated that one locus could control dozens of seed characteristics. Analysis of the loci overlap showed that this is typical for color traits and rarely occurred for seed size/shape traits. For a number of highly significant QTLs, we identified the physical location of their markers on the wheat chromosomes. Additionally, we demonstrated that the overlap between QTL regions was low for seed size/shape traits and high for coat color traits. Using the literature and KEGG data, we identified sets of genes in *Arabidopsis* and rice from the networks controlling seed size and color. This information along with the coordinates of the markers in the wheat genome was used for the prioritization of wheat genes within QTL regions. We identified 29 candidate genes from the seed size-related loci and 14 for genes from the loci associated with seed coat color. The genes we have identified as possible candidates associated with seed size/shape and color formation in the ITMI/MP can be further investigated in more detail using genetic and molecular methods to establish the mechanisms controlling these important traits. Our results demonstrate the complex nature of the genetic control of the wheat seed traits and the efficiency of the image analysis methods for obtaining novel QTLs for seed characteristics. 

## Figures and Tables

**Table 1:** List of candidate genes from QTLs associated with seed size/shape. Columns of the table contain QTL name (QTL), chromosome and position in cm (Chr/Pos), gene ID, KEGG orthogroup ID, KEGG orthogroup description, and EC number, if provided.

QTL	Chr/Pos	Gene ID	KO ID	Description	EC
Q.sA-3A	3A/155	*TraesCS3A03G0787100*	K09286	EREBP; EREBP-like factor	-
		*TraesCS3A03G0782400*	K09338	HD-ZIP; homeobox-leucine zipper protein	-
		*TraesCS3A03G0763900*	K00279	CKX; cytokinin dehydrogenase	EC:1.5.99.12
Q.sSo-4A	4A/305	*TraesCS4A03G1100100*	K19045	BB; E3 ubiquitin-protein ligase BIG BROTHER and related proteins	EC:2.3.2.27
Q.sA-2B.2	2B/208	*TraesCS2B03G0313000*	K09286	EREBP; EREBP-like factor	-
Q.sCi-2B ^c^; sSo-2B ^c^	2B/129	*TraesCS2B03G1115600*	K11593	ELF2C, AGO; eukaryotic translation initiation factor 2C	-
		*TraesCS2B03G1114800*	K11593	ELF2C, AGO; eukaryotic translation initiation factor 2C	-
		*TraesCS2B03G1105600*	K09422	MYBP; transcription factor MYB, plant	-
		*TraesCS2B03G1109700*	K09286	EREBP; EREBP-like factor	-
		*TraesCS2B03G1116700*	K11593	ELF2C, AGO; eukaryotic translation initiation factor 2C	-
		*TraesCS2B03G1104200*	K11593	ELF2C, AGO; eukaryotic translation initiation factor 2C	-
		*TraesCS2B03G1109900*	K09286	EREBP; EREBP-like factor	-
		*TraesCS2B03G1106500*	K09338	HD-ZIP; homeobox-leucine zipper protein	-
Q.sW-2D	2D/74	*TraesCS2D03G0133000*	K09422	MYBP; transcription factor MYB, plant	-
		*TraesCS2D03G0143000*	K09602	OTUB1; ubiquitin thioesterase protein OTUB1	EC:3.4.19.12
		*TraesCS2D03G0143400*	K09602	OTUB1; ubiquitin thioesterase protein OTUB1	EC:3.4.19.12
Q.sCi-2D ^g^; Q.sRo-2D ^g^	2D/58	*TraesCS2D03G0107400*	K09602	ubiquitin thioesterase protein OTUB1	EC:3.4.19.12
		*TraesCS2D03G0107900*	K09602	ubiquitin thioesterase protein OTUB1	EC:3.4.19.12
sSo-7D.2	7D/141	*TraesCS7D03G1008800*	K09286	EREBP; EREBP-like factor	-
		*TraesCS7D03G0987700*	K09338	HD-ZIP; homeobox-leucine zipper protein	-
		*TraesCS7D03G0983800*	K09286	EREBP; EREBP-like factor	-
		*TraesCS7D03G0972400*	K08869	ADCK, ABC1; aarF domain-containing kinase	-
		*TraesCS3A03G0763900*	K00279	CKX; cytokinin dehydrogenase	EC:1.5.99.12
sL-7D ^bb^; sRo-7D ^bb^	7D/287	*TraesCS7D03G1260500*	K20547	CHIB; basic endochitinase B	EC:3.2.1.14
		*TraesCS7D03G1260300*	K20547	CHIB; basic endochitinase B	EC:3.2.1.14
		*TraesCS7D03G1260400*	K20547	CHIB; basic endochitinase B	EC:3.2.1.14
		*TraesCS7D03G1286900*	K11593	ELF2C, AGO; eukaryotic translation initiation factor 2C	-
		*TraesCS7D03G1260600*	K20547	CHIB; basic endochitinase B	EC:3.2.1.14
		*TraesCS7D03G1287400*	K11593	ELF2C, AGO; eukaryotic translation initiation factor 2C	-

**Table 2:** List of candidate genes from QTLs associated with seed shell color. Columns of the table contain QTL name (QTL), chromosome and position in cm (Chr/Pos), gene ID, KEGG orthogroup ID, KEGG orthogroup description, EC number, KEGG pathway ID and description.

Trait	Chr/Pos	Gene ID	KO ID	KO Description	EC	KEGG Pathway ID	KEGG Pathway Description
Q.YCrCb_dCCr_1-2A.3	2A/196	*TraesCS6A03G0725700*	K09840	NCED; 9-cis-epoxycarotenoid dioxygenase	EC:1.13.11.51	map00906	Carotenoid biosynthesis
		*TraesCS2A03G0158600*	K22772	FG2; flavonol-3-O-glucoside L-rhamnosyltransferase	EC:2.4.1.159	map00944	Flavone and flavonol biosynthesis
		*TraesCS6A03G0953500*	K13065	HCT; shikimate O-hydroxycinnamoyltransferase	EC:2.3.1.133	map00941	Flavonoid biosynthesis
		*TraesCS2A03G0099800*	K13066	COMT; caffeic acid 3-O-methyltransferase/acetylserotonin O-methyltransferase	EC:2.1.1.68; 2.1.1.4	map00380	Tryptophan metabolism
Q.Lab_dCb_3-3A ^i^*	3A/195	*TraesCS3A03G0925800*	K01904	4CL; 4-coumarate--CoA ligase	EC:6.2.1.12	map00940	Phenylpropanoid biosynthesis
		*TraesCS3A03G0925900*	K01904	4CL; 4-coumarate--CoA ligase	EC:6.2.1.12	map00940	Phenylpropanoid biosynthesis
Q.RGB_dCB_1-3B.1	3B/269.179	*TraesCS3B03G1115600*	K12355	REF1; coniferyl-aldehyde dehydrogenase	EC:1.2.1.68	map00940	Phenylpropanoid biosynthesis
HSV_dCH_1-3B ^m^**	306.179	*TraesCS3B03G1278200*	K01904	4CL; 4-coumarate--CoA ligase	EC:6.2.1.12	map00940	Phenylpropanoid biosynthesis
HSV_dCH_3-6A ^t^***	6A/246	*TraesCS6A03G0725000*	K09843	CYP707A; (+)-abscisic acid 8’-hydroxylase	EC:1.14.14.137	map00906	Carotenoid biosynthesis
		*TraesCS6A03G0741000*	K00021	HMGCR; hydroxymethylglutaryl-CoA reductase (NADPH)	EC:1.1.1.34	map00900	Terpenoid backbone biosynthesis
Q.YCrCb_dCCr_1-6A	6A/340	*TraesCS6A03G0953500*	K13065	HCT; shikimate O-hydroxycinnamoyltransferase	EC:2.3.1.133	map00940	Phenylpropanoid biosynthesis
		*TraesCS2A03G0163500*	K00430	peroxidase	EC:1.11.1.7	map00940	Phenylpropanoid biosynthesis
		*TraesCS2A03G0164200*	K00430	peroxidase	EC:1.11.1.7	map00940	Phenylpropanoid biosynthesis
Lab_dCL_2-6B ^x^,****	6B/220	*TraesCS6B03G0367700*	K00430	peroxidase	EC:1.11.1.7	map00940	Phenylpropanoid biosynthesis

* Co-located QTL: Q.YCrCb_dCCb_3-3A ^i^. ** Co-located QTLs: HSV_dCH_3-3B ^m^; HSV_dCS_1-3B ^m^; HSV_dCV_1-3B ^m^; HSV_dCV_3-3B ^m^; Lab_dCb_1-3B ^m^; Lab_dCb_2-3B ^m^; Lab_mb-3B ^m^; RGB_dCR_2-3B ^m^; YCrCb_dCCb_1-3B ^m^; YCrCb_dCCb_2-3B ^m^; YCrCb_dCCr_1-3B ^m^; YCrCb_mCb-3B ^m^. *** Co-located QTL: HSV_dCV_3-6A ^t^; Lab_ma-6A ^t^. **** Co-located QTLs: Lab_dCL_3-6B ^x^; Lab_mb-6B ^x^; RGB_dCG_2-6B ^x^; RGB_dCR_2-6B ^x^; YCrCb_dCCb_2-6B ^x^; YCrCb_dCY_2-6B ^x^; YCrCb_dCY_3-6B ^x^.

## Data Availability

Not applicable.

## Supplementary Materials

The following supporting information can be downloaded at https://www.mdpi.com/article/10.3390/plants11162105/s1: Figure S1: PCA biplot of seed size/shape and color traits of ITMI/MP performed using seed coat traits (mean seven values for seed size/shape and 12 values for color components of four-color spaces); Figure S2: Examples of seed images used for digital phenotyping; Table S1: Complete list of QTLs identified through composite interval mapping. Left and right flanking markers linked to QTL are also provided along with LOD, PVE%, and additive effect (+ = provided by Opata and -= provided by W7984 parent). Similar asterisks (also highlighted in similar color) indicate likely identical loci. Markers highlighted in red are involved in multiple QTLs. Table S2: Pairs of epistatic quantitative trait loci detected in the ITMI/MP. Table S3: List of 197 (42 from rice and 155 from Arabidopsis) genes involved in molecular processes of the grain development. Input Gene ID = Gene ID from review papers; KEGG Gene ID = GID for gene in KEGG database; NA: not found in KEGG; KEGG Gene Name = Gene name in KEGG database and KEGG Orthology ID = Orthogroup ID in KEGG database. Table S4: Pathway ID and KEGG orthologous groups. Pathway KEGG = pathway ID and KEGG orthology group = Orthogroup ID in KEGG database. Table S5: List of QTLs used in gene prioritization. Table S6: List of 823 genes associated with grain color.
