# Supplementary material for: QTL Analysis for Bread Wheat Seed Size, Shape and Color Characteristics Estimated by Digital Image Processing
Source: Plants (Basel). 2022 Aug 12;11(16):2105. doi: 10.3390/plants11162105 (PMC9414870; doi:10.3390/plants11162105)
Supplement: Supplementary file 1 [file plants-11-02105-s001.zip › Figure S1.pdf]

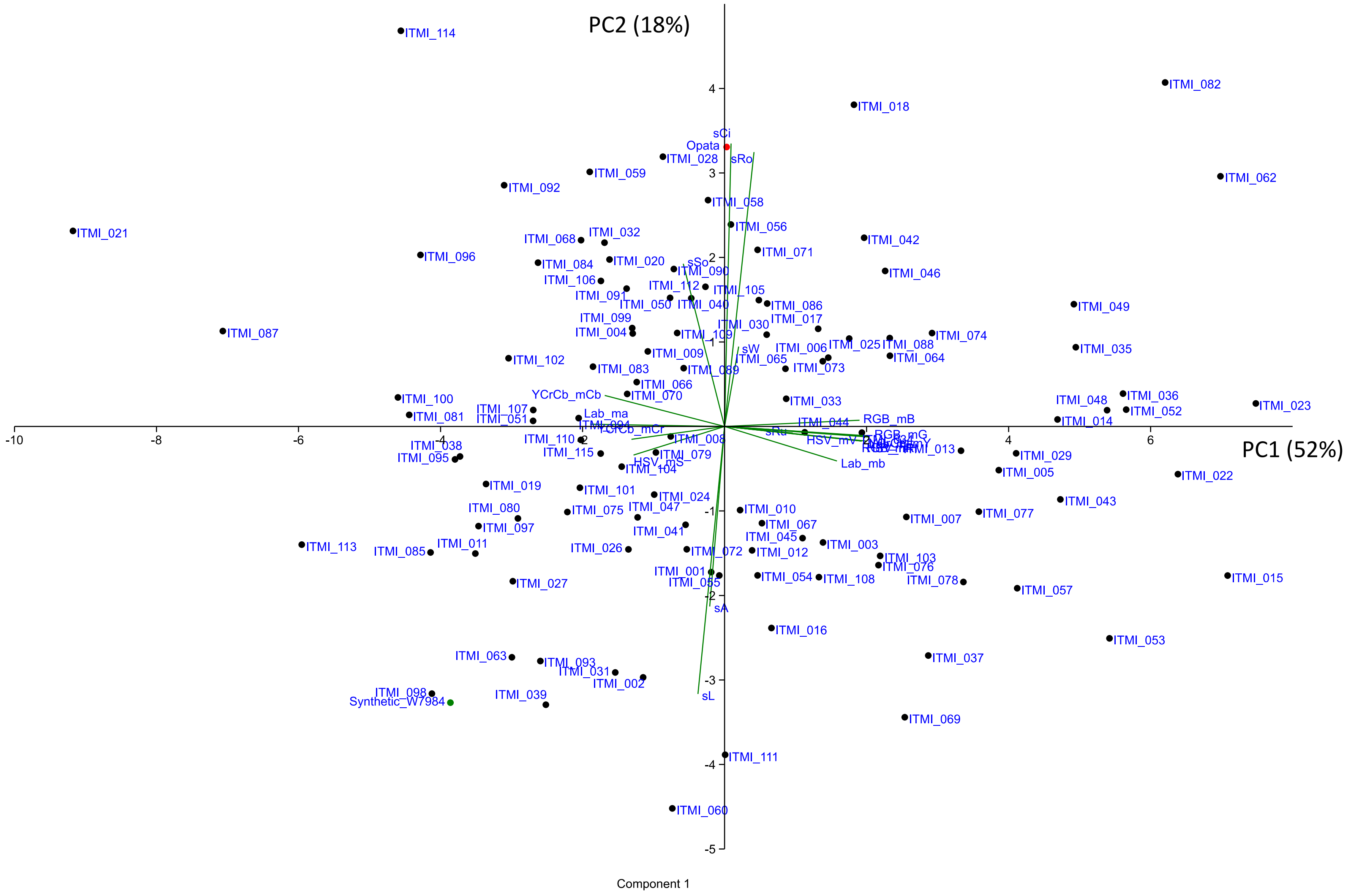

Figure S1. PCA biplot of ITMI/MP's seed size/shape and color seed traits (mean values for 7 size/shape and 12 color components of four color spaces). Ellipses represent seed size and shape for some contrast genotypes. Parent genotypes are shown by green (Synthetic_W7984) and red (Opata) dots. PC1, PC2 denotes principal components 1 and 2, percentage of variance explained shown in parentheses.
