# Supplementary figures and images for: QTL Analysis for Bread Wheat Seed Size, Shape and Color Characteristics Estimated by Digital Image Processing
Source: Plants (Basel). 2022 Aug 12;11(16):2105. doi: 10.3390/plants11162105 (PMC9414870; doi:10.3390/plants11162105)

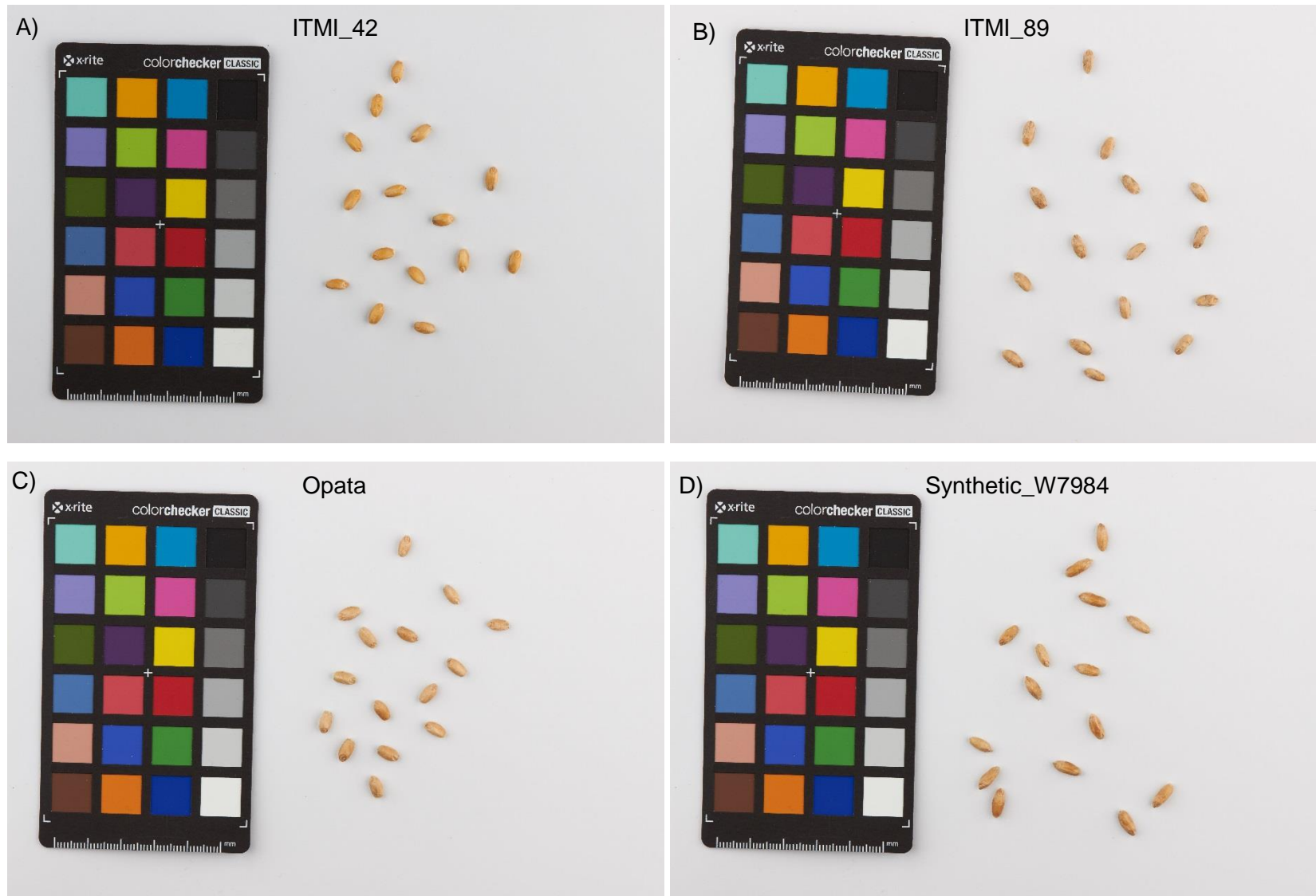

**Figure S2.** Examples of seed images used for digital phenotyping: (A) ITMI\_42; (B) ITMI\_89, (C) Opata, (D) Synthetic\_W7984.

Supplement: Supplementary file 1 [file plants-11-02105-s001.zip › Figure S2.pdf]
